# Factors associated with euphoria in a large subset of cases using propofol sedation during gastrointestinal endoscopy

**DOI:** 10.3389/fpsyt.2023.1001626

**Published:** 2023-04-27

**Authors:** Kaixuan Zhao, Ning Yang, Jingli Yue, Ying Han, Xiaoxiao Wang, Ning Kang, Tianhao Zhang, Xiangyang Guo, Mao Xu

**Affiliations:** ^1^Department of Anesthesiology, Peking University Third Hospital, Beijing, China; ^2^Department of Psychiatry and Clinical Psychology, Peking University Sixth Hospital, Beijing, China; ^3^National Institute of Drug Dependence and Beijing Key Laboratory of Drug Dependence, Peking University, Beijing, China; ^4^Research Center of Clinical Epidemiology, Peking University Third Hospital, Beijing, China

**Keywords:** propofol, euphoria, sedative, drug abuse and addictive potential, gastrointestinal endoscopy

## Abstract

**Background:**

The utilization of Propofol, a widely used intravenous sedative or anesthetic, is characterized by its quick onset, predictable control, and fleeting half-life during both general anesthesia and intensive care unit sedation. Recent evidence, however, has highlighted propofol’s propensity to induce euphoria, particularly in patients undergoing painless procedures such as gastrointestinal or gastric endoscopy. Given its widespread use in patients undergoing such procedures, this study aims to investigate the clinical evidence and factors that may influence propofol-induced euphoria in these settings.

**Methods:**

The Addiction Research Center Inventory-Chinese Version (ARCI-CV) scale was administered to 360 patients undergoing gastric or gastrointestinal endoscopy using propofol as a sedative. Patient characteristics including past medical history, depression, anxiety, alcohol abuse, and sleep disturbance were recorded through history taking and assessment using various questionnaires prior to the examination. The euphoric and sedative statuses were assessed at 30 min and 1 week post-examination.

**Results:**

The experimental results of a survey of 360 patients who underwent gastric or gastrointestinal endoscopy using propofol showed that the mean Morphine–Benzedrine Group (MBG) score before the procedure and after 30 min of the procedure was 4.23 and 8.67, respectively. The mean Pentobarbital–Chlorpromazine–Alcohol Group (PCAG) score before the procedure and after 30 min of the procedure was 3.24 and 6.22, respectively. These results showed that both MBG and PCAG scores increased significantly after the procedure. Certain factors, such as dreaming, propofol dose, duration of anesthesia, and etomidate dose, were all correlated with MBG both at 30 min and 1 week after the examination. In addition, etomidate had an effect of decreasing MBG scores and increasing PCAG scores both at 30 min and 1 week after the examination.

**Conclusion:**

Taken together, propofol may elicit euphoria and potentially contribute to propofol addiction. There are several risk factors for the development of propofol addiction, including dreaming, propofol dose, duration of anesthesia, and etomidate dose. These findings suggest that propofol may have a euphoric effect and may have the potential for drug addiction and abuse.

## 1. Introduction

Propofol is a widely utilized intravenous sedative or anesthetic characterized by its quick onset, manageable control, and fleeting half-life (1). It is used for procedural sedation, monitored anesthesia care, and as an induction agent for general anesthesia. However, propofol dependence and abuse have gradually attracted extensive attention after pop star Michael Jackson died in 2009 due to propofol intoxication (2). Tezcan et al. reported that the euphoric effect score (Morphine–Benzedrine Group, MBG)of propofol reach about 9.8, which was quite high (3). The first use produces pleasure, relaxation, and euphoria, making it difficult to stop (4). Current case reports and epidemiological studies showed that propofol abuse is relatively concentrated in the United States, Germany, South Korea, and other countries (4–6). In the United States, up to 3% of anesthesia medical workers have a dependence on and abuse propofol (7). In a survey of South Korean medical staff, 11.5% of participants reported the abuse of propofol among colleagues (8). From 2000 to 2011, South Korea reported 20 deaths caused by propofol abuse, including 14 doctors, nurses, and hospital managers (8). In addition, propofol was detected in 131 cases out of 14,673 autopsied cases within 6 years in South Korea (6). The abuse of propofol seriously endangers the health of the public and medical personnel, especially anesthesiologists and nurses. Propofol also showed psychoactive effects similar to other addictive drugs in healthy adult volunteers (9).

Clinically, propofol-induced euphoria is more common in patients undergoing short or painless surgeries, such as gastric or gastrointestinal endoscopy (3, 10–12). However, to the best of our knowledge, limited clinical trials have investigated the euphoria and abuse potential for propofol after endoscopic procedures. As the aging population in China continues to grow, the number of gastric or gastrointestinal endoscopies is increasing. There is limited research on the prevalence of propofol euphoria in endoscopy patients in China. Therefore, it is imperative to investigate the frequency and number of euphoric side effects specific to gastric or gastrointestinal endoscopy under propofol anesthesia.

This study explored the potential for propofol abuse based on its euphoric effects assessed by the Morphine–Benzedrine Group (MBG) scale during endoscopic procedures by current clinical guidelines (10). For example, opioid analgesics, such as low-dose fentanyl or sufentanil, are widely used. Etomidate is sometimes applied for the induction of anesthesia to relieve possible respiratory depression and injection pain. The main purpose of this study was to observe the euphoria induced by propofol sedation during gastric or gastrointestinal endoscopy in clinical settings. This study may make anesthesiologists and gastroenterologists pay more attention to this clinical phenomenon and provide a reference for better guiding clinical medication strategies.

## 2. Materials and methods

### 2.1. Ethical statement

This study was conducted in accordance with the Declaration of Helsinki and approved by the Ethics Committee of Peking University Third Hospital (Grant number IRB00006761-m2021106). This prospective observational study was also registered in the Chinese Clinical Trial Registry (November 18^th^, 2021, registry number ChiCTR2100046127). All participants were recruited from Peking University Third Hospital (Beijing, China) from November 2021 to May 2022. Written informed consent was obtained from each patient. The psychological assessment was carried out under the guidance of a qualified psychiatrist Yue Jingli whose certification had been submitted to the Ethics Committee for verification. To avoid bias and possible artificially induced drug dependence, the present research was advised by the Ethics Committee not to discuss the possibility of euphoria with patients in advance. Under the requirements of the local ethics committee, the patients were informed that they were participating in a study of the non-anesthetic effects of Propofol, which is scientifically and essentially accurate, but the euphoric effect of propofol was not specifically emphasized in the informed consent form. In this consent, we used alternative language to refer to addiction, such as “emotional changes” rather than using terms that may imply seeking drugs or hint at addiction. A translated copy of the informed consent can be found in the Supplementary material.

### 2.2. Study population

Adult patients with American Society of Anesthesiologists Classification grades 1–2 for gastric or gastrointestinal endoscopy under general anesthesia in our hospital were eligible for inclusion, but patients with one of the following conditions were excluded: (a) a history of psychiatric disorders, such as schizophrenia, bipolar disorder, personality disorder, etc.; (b) a known hypersensitivity reaction to any drugs, such as allergy to propofol or benzodiazepines, or intolerance to them; (c) a recent surgery or major trauma within 14 days prior to the experiment; (d) younger than 18 or over 80 years old. All patients were given written informed consent for participation in the research project. After obtaining informed consent, each participant was presented with a signed copy of the document and provided with an exhaustive explication of the aim and nature of the present investigation.

### 2.3. Objectives

The primary outcome of the study was to evaluate the frequency and extent of euphoria and residual sedation after gastroenteroscopy under propofol anesthesia and assess the residual memory of such effects after 1 week. The secondary objective was to analyze how the patients’ basic situations and psychological parameters before the examination (such as general characteristics, depression tendency, anxiety tendency, and sleep problems), as well as addictive behaviors (such as smoking and drinking), influence the euphoric feelings.

### 2.4. Schedule

A total of 360 patients undergoing gastric or endoscopy were recruited. Informed consent was obtained from all patients before they entered the trial. The patients each completed a questionnaire at least 1 h prior to endoscopy (T0). In the questionnaire, basic data were collected, including age, gender, height, weight, and education years. Before endoscopy (T1), all participants completed the Alcohol Use Disorders Identification Test (AUDIT), Pittsburgh Sleep Quality Index (PSQI), Beck Anxiety Inventory (BAI), and Center for Epidemiologic Studies Depression Scale (CES-D), Addiction Research Center Inventory (ARCI) scale, and smoking and past medical history evaluations. After patients were admitted to the operating room, electrocardiogram (ECG), mean arterial pressure, heart rate, and pulse oximetry (SpO_2_) were recorded, and the anesthesia time and auxiliary drugs used for anesthesia were also recorded. The Addiction Research Center Inventory (ARCI) scale was repeated 30 min after endoscopy (T2) and 1 week later (T3, by telephone call). They were told to recall how they felt at the time point when they woke up. Patients were asked briefly whether they had a dream and, if so, whether the dreams were pleasant at the same time (T2). In this study, the ARCI scores obtained before the procedure were labeled MBG-Before procedure (MBG-BP) and PCAG-Before procedure (PCAG-BP). The ARCI scores obtained 30 min after the procedure were labeled MBG-30 min and PCAG-30 min. The results of 1 week follow-up were labeled MBG-1 week and PCAG-1 week.

### 2.5. Anesthesia and surgical management

This clinical trial is a prospective observational cohort study. To avoid bias, the anesthetic procedure was not intervened by the study staff. The anesthesia was consistent with the methods recommended by current clinical guidelines (10).

Before gastrointestinal endoscopy, intravenous catheterization was performed in the anesthesia preparation room. Ringer’s lactate solution was slowly infused to maintain the usual intravenous access, and then the patient was transferred to the operating room to start the monitoring of non-invasive blood pressure, ECG, heart rate, and SpO_2_.

According to the patient’s age, past medical history, obesity, and other factors, the anesthesia practitioner decided whether to use etomidate during induction.

In all patients, a small dose of fentanyl or sufentanil was first administered in a single injection, usually 5 μg of sufentanil or 100 μg of fentanyl only during anesthesia induction.

Then, the patients were induced with 0.5–2.5 mg/kg propofol and/or 0–0.3 mg/kg etomidate. When etomidate was administered, the dose of propofol was reduced, or propofol was not used for induction.

Anesthesia was maintained using propofol continuous infusion with the infusion rate adjusted to maintain a moderate to deep sedation according to the American Society of Anesthesiologists (ASA) sedation/anesthesia classification (10). The gastrointestinal endoscopy was started when loss of the eyelash reflex was confirmed (10). The dose of propofol and the duration of anesthesia were recorded using an electronic infusion pump. If a patient developed complications during anesthesia, such as hypotension, body movement, and low oxygen saturation, they were recorded accordingly. In addition, anesthesiologists administered treatment in accordance with clinical recommendations. In this study, hypotension was defined as systolic blood pressure below 90 mmHg, and low oxygen saturation was defined as SpO_2_ below 90%.

### 2.6. Questionnaires

The Alcohol Use Disorders Identification Test (AUDIT), Pittsburgh Sleep Quality Index (PSQI), Beck Anxiety Inventory (BAI), and Center for Epidemiologic Studies Depression Scale (CES-D) were applied to assess drinking, sleeping, anxiety, and depression, respectively.

The AUDIT (Alcohol Use Disorders Identification Test) is a screening tool used to identify individuals who may be at risk for alcohol use disorders. The Cronbach’s alpha of the AUDIT (Alcohol Use Disorders Identification Test) is 0.83, indicating good reliability14.

The Pittsburgh Sleep Quality Index (PSQI) is a widely used self-report questionnaire that measures general sleep quality in general populations15 16. It has a Chinese version and a Cronbach’s alpha coefficient of 0.8315 16.

The Beck Anxiety Inventory (BAI) is a self-report measure of anxiety with 21 items. The Beck Anxiety Inventory (BAI) has good psychometric properties and a Cronbach’s alpha of 0.9217.

The CES-D has also been found to have good reliability in various studies conducted in different countries, including China, France, Armenia, and Hong Kong (11). In general populations, the Cronbach’s alpha coefficients of the CES-D is 0.87 (12).

The Addiction Research Center Inventory (ARCI) scale was developed by the National Institute of Mental Health Addiction Research Center (United States). It is mainly used to quantify the specific mental effects of various psychoactive substances with abuse potential. Subjects were asked to recall feelings following the use of a certain psychoactive substance and answered in response to the mood at that time. The Cronbach’s alpha for the ARCI scale has been found to be 0.86 in one study, indicating that it exhibits acceptable test–retest reliability (13).

The ARCI-CV is a set of scales that have been translated by several experts and tested for reliability and validity (14, 15). The ARCI-CV is composed of three components: the Morphine–Benzedrine Group (MBG) used to measure the euphoric effect of drugs, Pentobarbital–Chlorpromazine–Alcohol Group (PCAG) used to measure the sedation of drugs, and the Lysergic Acid Diethylamide scale (LSD) used to measure the psychotomimetic effect of drugs. The MBG subscale in the ARCI was used as a diagnostic tool to evaluate whether patients had euphoria and the severity of euphoria (15).

After endoscopy, we administered the MBG subscale in the ARCI-CV to 360 patients who used propofol as a sedative to rate euphoria and the PCAG subscale to rate residual sedation.

### 2.7. Statistics

Statistical analyses were performed with SPSS 26.0. We compared the average value of the survey results to determine whether there are any differences in the basic characteristics of the subjects (such as age or gender) and performed a multiple regression analysis of the results for possible factors such as age, gender, education, BMI, ASA, preoperative complications, duration of anesthesia, propofol infusion rate, induction dose, fentanyl dose, etomidate dose, intraoperative adverse reactions, smoking habits, drinking problems, depression tendency, anxiety tendency, and dream condition. Differences in dream conditions were analyzed using one-way repeated measures ANOVA. MBG and PCAG scores were analyzed according to gender to determine whether this factor was related to MBG and PCAG scores. MBG and PCAG scores results obtained 30 min and 1 week postoperatively were assessed using correlation tests and paired samples t-tests. PASS 15.0 software was used to estimate the sample size of multiple regression analysis. When the test efficiency was 0.9 at an alpha error of 0.05 and the independent variables were estimated to be 20, the sample size was supposed to be at least 149.

## 3. Results

### 3.1. Demographic characteristics of subjects

**Figure 1:**
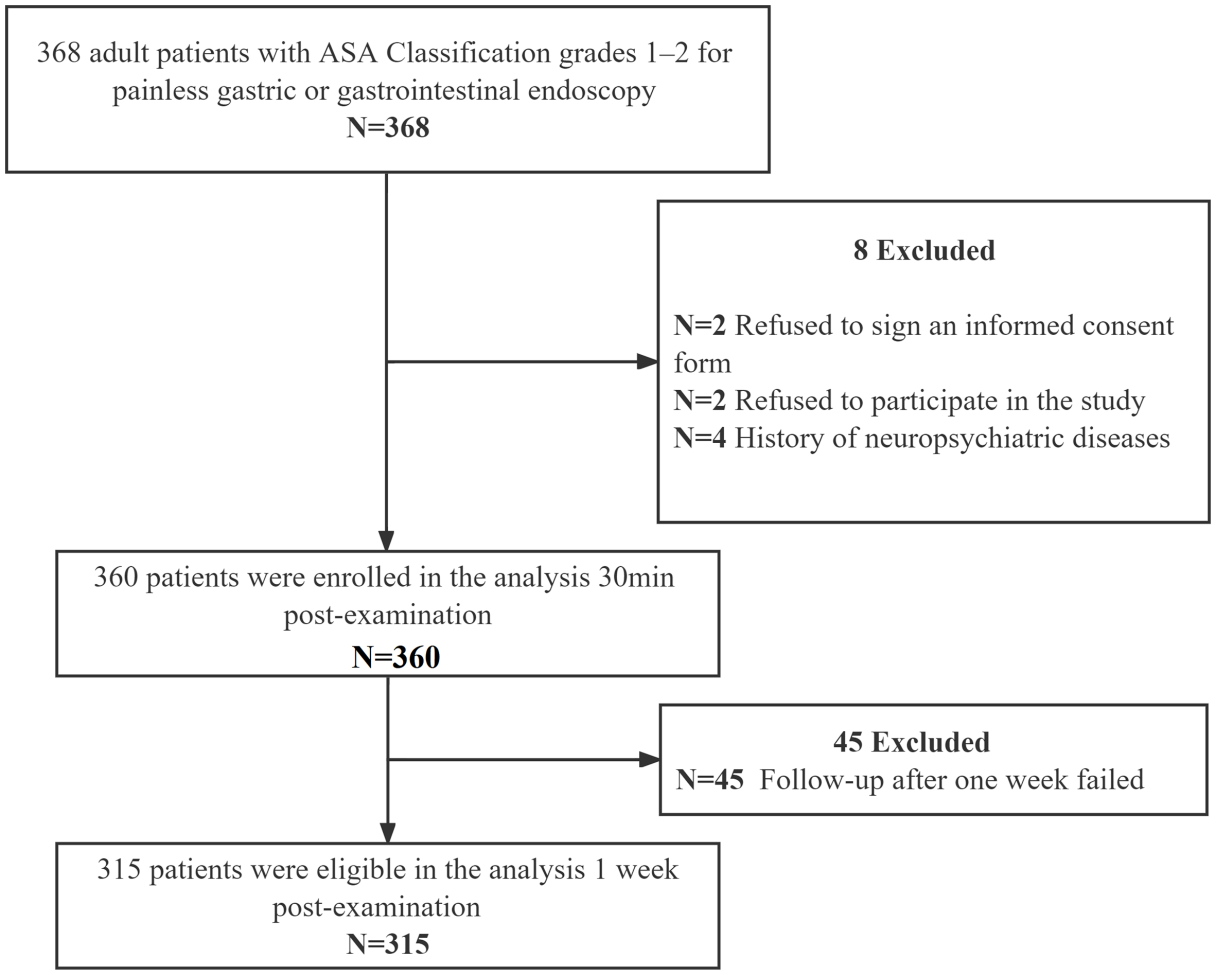
Study flow chart. In total, 368 patients were initially screened for the study, and 360 patients were included in the data analysis 30 min post-examination as well as 315 patients were recruited in the data analysis 1 week post-examination. ASA, American Society of Anesthesiologists.

In total, 368 patients were initially screened for the study. 360 patients and 315 patients were included in the data analysis at 30 min and 1-week post-examination, respectively, (Figure 1). The demographic characteristics are shown in Table 1.

**Table 1:** Demographic characteristics.

Items	Categories	*N* or Value
Education	Bachelor	199 (55.28)
	Doctor’s degree	43 (11.94)
	Junior college	30 (8.33)
	Junior high school	17 (4.72)
	Master’s degree	60 (16.67)
	Senior high school and higher vocational education	11 (3.06)
Occupation	Retired	2 (0.56)
	Students	22 (6.11)
	Urban incumbency	280 (77.78)
	Urban unemployment	4 (1.11)
	farmer	4 (1.11)
	retired	4 (13.33)
Total		360
Types of examinations	Colonoscopy	23 (6.39)
	Gastroenteroscope	295 (81.94)
	Gastroscope	42 (11.67)
Preoperative complications	After Operation for Thyroid	3 (0.83)
	Allergic constitution	2 (0.56)
	Carotid artery stenosis	2 (0.56)
	Diabetes	6 (1.67)
	Hashimoto thyroiditis	1 (0.28)
	Hyperlipidemia	19 (5.28)
	Hypertension	33 (9.17)
	Hypertension/Hyperlipidemia	1 (0.28)
	Hypertension&Hypothyroidism& Hypothyroidism	3 (0.83)
	Hypothyroidism	1 (0.28)
	No complications	266 (73.89)
	Obesity	5 (1.39)
	Previous history of propofol anesthesia	6 (1.67)
	Thyroid nodule	2 (0.56)
	Hypotension	4 (1.11)
	Hypothyroidism	5 (1.39)
	Mitral stenosis	1 (0.28)
Intraoperative adverse reactions	Arrhythmia	2 (0.56)
	Body movement	37 (10.28)
	Hypertension	11 (3.06)
	Hypotension	7 (1.95)
	Low oxygen saturation	28 (7.78)
	Multiple complications	1 (0.28)
	No complications	273 (75.83)
	Hypertension/Body movement	1 (0.28)
Age(years,18-73)		43.97 ± 12.56
Duration of education (years)		16.21 ± 2.35
BMI		22.87 ± 3.22
Duration of anesthesia (minutes)		17.96 ± 9.63
Total propofol infusion (mg)		171.52 ± 90.94
Propofol dose (mg/kg)		2.72 ± 1.47
Propofol infusion rate (mg/kgmin)		0.16 ± 0.08
Propofol induction dose (mg/h)		61.88 ± 37.32
Propofol infusion speed (mL/h)		33.67 ± 9.42
Sufentanyl (mcg/kg)		0.03 ± 0.05
*Sufentanyl (mcg/kg) (*n* = 112)		0.10 ± 0.04
Fentanyl (mcg/kg)		1.07 ± 0.78
*Fentanyl (mcg/kg) (*n* = 244)		1.57 ± 0.32
Etomidate dose (mg/kg)		0.09 ± 0.11
Smoking		0.92 ± 3.84

Categorical variables were presented as number of cases (percentage) and continuous variables are expressed as mean ± standard deviation (SD). *Because patients received only one of these drugs, fentanyl or sufentanil, this term was calculated for patients who used this drug only. BMI, Body mass index.

### 3.2. Measurement of differences between genders

Considering that this study included patients of both genders, we explored whether gender is an important influencing factor. The results showed that the average MBG score before the examination among males was 4.10 ± 1.20 and females was 4.32 ± 1.26, with no significant difference. The mean PCAG score before the examination among male was 3.36 ± 1.98 and female was 3.16 ± 1.80, also without any statistical significance. The results showed that the average MBG score 30 min after recovery (MBG-30 min) in 360 patients who underwent gastroenteroscopy with propofol was 8.67, and there was no significant gender difference. The average PCAG-30 min among males was 5.18 ± 3.09, while it was 6.22 ± 3.17 for females (*p* = 0.002) (Table 2). After 1 week of follow-up, the average MBG score (MBG-1 week) was 8.81, and the gender difference was not significant. The average PCAG-1 week among males was 4.72 ± 2.86, and the average PCAG-1 week among females was 5.47 ± 3.46, showing a statistically significant difference (*p* = 0.043) (Table 2). This result suggests that men and women show similar levels of euphoria from propofol. However, the PCAG scores of female patients at 30 min and 1 week after the procedure were significantly higher than those of male patients. These findings suggest that propofol induces more sedation in females compared to males.

**Table 2:** Difference between males and females values are expressed as mean ± standard deviation (SD).

	Total	Gender		*t*	*p*
		Male (*n* = 143)	Female (*n* = 217)		
Age (yrs)	43.97 ± 12.56	44.29 ± 11.39	43.76 ± 13.29	0.407	0.694
Duration of Education (mins)	16.21 ± 2.35	16.47 ± 2.33	16.03 ± 2.36	1.726	0.085
Height (cm)	166.74 ± 8.26	173.62 ± 6.83	162.20 ± 5.54	16.700	0.000^***^
Weight (kg)	63.91 ± 12.2	73.30 ± 10.94	57.73 ± 8.47	14.410	0.000^***^
BMI	22.87 ± 3.22	24.26 ± 2.99	21.94 ± 3.04	7.125	0.000^***^
MBG-BP	4.23 ± 1.26	4.10 ± 1.20	4.32 ± 1.26	−1.595	0.112
MBG-30 min	8.67 ± 4.69	8.43 ± 4.84	8.81 ± 4.58	0.498	0.481
MBG-1 week	8.81 ± 4.59	8.69 ± 4.49	8.81 ± 4.59	0.143	0.705
PCAG-BP	3.24 ± 1.88	3.36 ± 1.98	3.16 ± 1.80	0.967	0.334
PCAG-30 min	6.22 ± 3.17	5.18 ± 3.09	6.22 ± 3.17	9.376	0.002^**^
PCAG-1 week	5.17 ± 3.25	4.72 ± 2.86	5.47 ± 3.46	4.115	0.043^*^

MBG, Morphine-amphetamine subscale to describe euphoria; PCAG, pentobarbital chlorpromazine ethanol subscale to describe residual sedation; MBG-BP, PCAG-BP were obtained before examine; MBG-30 min, PCAG-30 min were obtained 30 min after recovery of endoscopic examination; MBG-1 week, PCAG-1 week were obtained a week after the anesthesia.

^*^p < 0.05, ^**^p < 0.01, ^***^p < 0.001.

### 3.3. MBG scores and influencing factors

**Figure 2:**
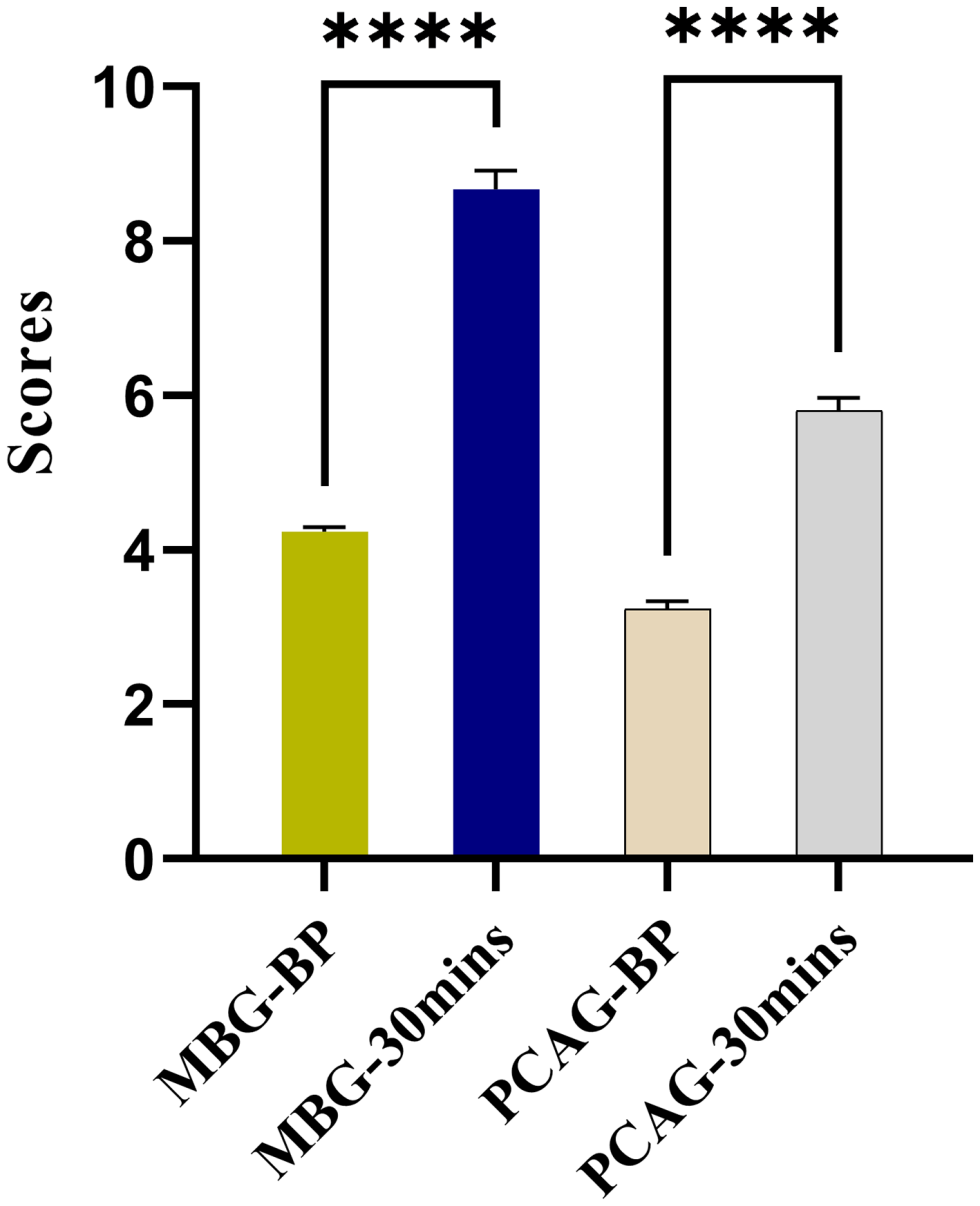
Experimental results of MBG and PCAG scores pre-procedure vs. post-procedure on 360 patients undergoing gastric or gastrointestinal endoscopy using propofol as a sedative. ^****^ *p* < 0.0001.

The experimental results of a survey of 360 patients who underwent gastric or gastrointestinal endoscopy using propofol showed that the mean MBG score before and after 30 min of the procedure was 4.23 and 8.67, respectively. The mean PCAG score before and after 30 min of the procedure was 3.24 and 6.22, respectively (Table 1 and Figure 2). These results showed that both MBG and PCAG scored improved significantly after the procedure.

**Figure 3:**
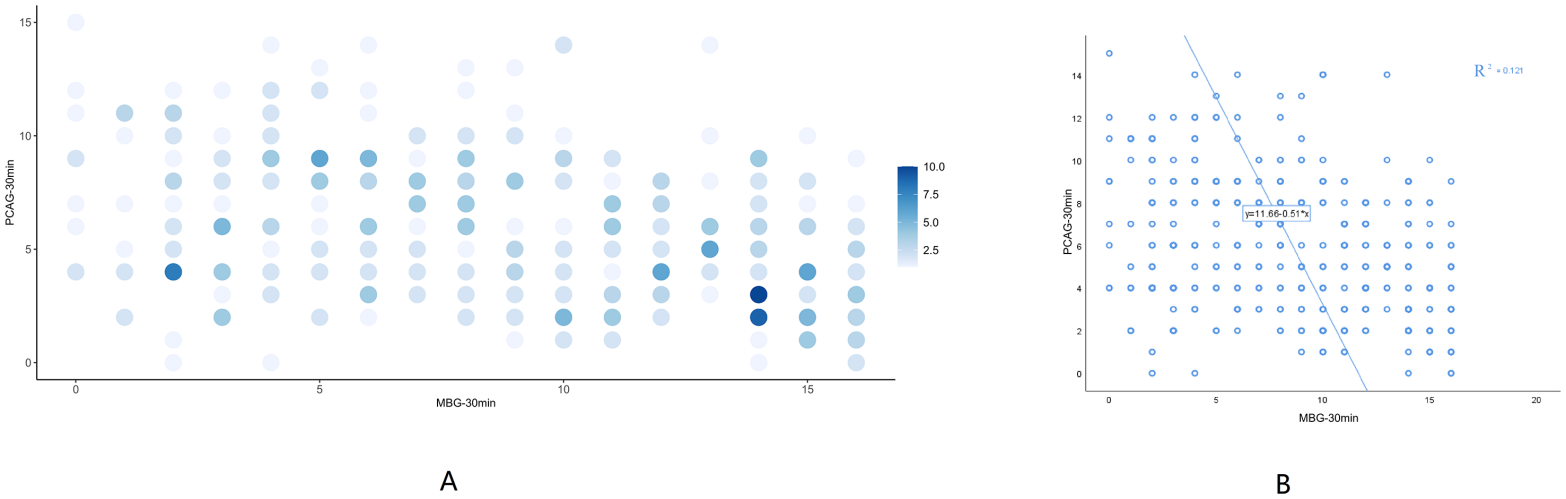
The correlation between MBG-30 min and PCAG-30 min. **(A)** Density scatter plots of MBG-30 min and PCAG-30 min. The deeper the density of points, the more MBG and PCAG are overlapped. **(B)** Scatter plots with regression line of MBG-30 min and PCAG-30 min. Linear regression analysis with PCAG-30 min as the independent variable and MBG-30 min as the dependent variable yielded the regression formula MBG-30 min =11.656–0.514 ^*^PCAG-30 min with a model R-square value of 0.121, implying that PCAG-30 min could explain 12.1% of the variation in MBG-30 min causes. F-test of the model revealed that the PCAG-30 min must have an impact on the MBG-30 min. (*F* = 49.421, *p* < 0.001). MBG, Morphine-amphetamine subscale; PCAG, pentobarbital chlorpromazine ethanol subscale. Data were presented as mean ± SEM.

To further explore the factors affecting euphoria, we examined MBG scores and possible influencing factors. After excluding co-linear variables, linear regression analysis was performed with MBG as the dependent variable and the possible influencing factors as the independent variables (Table 3). Propofol infusion rate (mg/kg/min) (*p* = 0.004), dream condition (*p* < 0.001), and AUDIT (*p* = 0.048) would result in significantly increased MBG-30 min scores. We also found a negative linear correlation between MBG and PCAG (*R*^2^ = 0.121) (Figure 3).

**Table 3:** Multiple linear regression analysis of MBG-30 min and related factors.

	Unstandardized coefficients		Standardized coefficients	*t*	*p*	VIF
	*B*	Std. Error	*Beta*			
Constant	6.152	4.731	–	1.300	0.194	–
Age	−0.003	0.021	−0.008	−0.135	0.893	1.403
Gender	1.249	0.587	0.130	2.127	0.054^†^	1.653
Duration of education	−0.087	0.103	−0.044	−0.848	0.397	1.168
BMI	−0.129	0.090	−0.088	−1.425	0.155	1.699
ASA	1.072	0.576	0.110	1.859	0.064^†^	1.542
Duration of anesthesia (minutes)	0.044	0.026	0.090	1.678	0.094^†^	1.278
Propofol infusion rate (mg/kg/min)	11.146	3.809	0.183	2.927	0.004^**^	1.728
Induction dose (mg)	−0.009	0.008	−0.075	−1.154	0.249	1.847
Infusion speed (mL/h)	0.018	0.027	0.036	0.674	0.500	1.264
Sufentanyl dose (μg/kg)	−14.346	8.849	−0.162	−1.621	0.106	4.413
Fentanyl dose (μg/kg)	−1.122	0.614	−0.186	−1.828	0.069^†^	4.540
Etomidate dose (mg/kg)	−0.609	2.472	−0.015	−0.247	0.805	1.580
Intraoperative adverse reactions	0.017	0.136	0.006	0.127	0.899	1.092
Smoking	0.079	0.065	0.065	1.214	0.226	1.262
Dream Condition	0.965	0.264	0.183	3.653	0.000^***^	1.101
AUDIT	0.196	0.099	0.112	1.982	0.048^*^	1.416
BAI	0.057	0.082	0.042	0.695	0.488	1.608
PSQI	0.094	0.071	0.074	1.320	0.188	1.373
CESD	−0.057	0.071	−0.047	−0.806	0.421	1.476

Propofol infusion rate (mg/kg/min), dream condition, AUDIT would result in a significant positive MBG effect relationship. D-W: 2.173. ^†^*p* < 0.1, ^*^p < 0.05, ^**^p < 0.01, ^***^p < 0.001. Adj R^2^ = 0.190. BMI, Body Mass Index; ASA, American Society of Anesthesiologists Classification (ASA Class); AUDIT, The Alcohol Use Disorders Identification Test; BAI, Beck Anxiety Inventory; PSQI, Pittsburgh Sleep Quality Index; CESD, Center for Epidemiologic Studies Depression Scale; VIF, Variance inflation factor.

To avoid bias caused by the subjective factors of the experimenter and the patient’s inability to answer the question correctly because of residual sedation effects, we followed up with the patients after 1 week. Follow-up was conducted by filling out a standard online questionnaire or telephone calls. Ninety-nine online questionnaires were collected, and the others were followed up by telephone. Forty-five patients were not followed up because of inevitable issues. The results were obtained for MBG-1 week, the same analysis was performed, and the results showed that duration of anesthesia (minutes) (*p* = 0.017), propofol infusion rate (mg/kg/min) (*p* = 0.001), and dream condition (*p* = 0.003) had a significant positive impact on MBG-1 week (Table 4).

**Table 4:** Multiple linear regression analysis of MBG-1 week and related factors.

	Unstandardized coefficients		Standardized coefficients	*t*	*p*	VIF
	*B*	Std. Error	*Beta*			
Constant	3.299	5.079	–	0.650	0.516	–
Age	−0.018	0.023	−0.048	−0.771	0.442	1.480
Gender	0.740	0.622	0.079	1.190	0.235	1.673
Duration of education	−0.072	0.109	−0.037	−0.659	0.510	1.168
BMI	−0.052	0.104	−0.036	−0.497	0.620	1.961
ASA	1.156	0.613	0.121	1.886	0.060^†^	1.563
Duration of anesthesia (minutes)	0.065	0.027	0.141	2.409	0.017^*^	1.292
Propofol infusion rate (mg/kg/min)	13.140	3.919	0.229	3.353	0.001^**^	1.763
Induction dose (mg)	−0.002	0.009	−0.017	−0.242	0.809	1.927
Infusion speed (mL/h)	0.005	0.027	0.011	0.195	0.846	1.261
Sufentanyl dose (μg/kg)	−16.239	9.591	−0.186	−1.693	0.092	4.568
Fentanyl dose (μg/kg)	−1.083	0.663	−0.182	−1.633	0.103	4.703
Etomidate dose (mg/kg)	9.751	11.820	0.246	0.825	0.410	33.619
Intraoperative adverse reactions	−0.017	0.147	−0.006	−0.116	0.908	1.114
Smoking	0.009	0.071	0.007	0.129	0.897	1.190
Etomidate	−0.210	0.194	−0.320	−1.078	0.282	33.285
Dream condition	0.829	0.280	0.162	2.962	0.003^**^	1.129
AUDIT	0.118	0.104	0.067	1.134	0.258	1.336
BAI	0.126	0.089	0.090	1.424	0.155	1.527
PSQI	0.133	0.077	0.103	1.734	0.084^†^	1.333
CESD	−0.112	0.074	−0.092	−1.509	0.132	1.397

The summary analysis showed that: Preoperative complications, Duration of anesthesia (minutes), Propofol infusion rate (mg/kg/min), Dream Condition had a significant positive impact on MBG-1 week.VIF, The variance inflation factor is a measure of the severity of complex (multiple) collinearities in a multiple linear regression model. The value of VIF less than 5 indicates no collinearity. BMI, Body Mass Index; ASA, American Society of Anesthesiologists Classification (ASA Class); AUDIT, The Alcohol Use Disorders Identification Test; BAI, Beck Anxiety Inventory; PSQI, Pittsburgh Sleep Quality Index; CESD, Center for Epidemiologic Studies Depression Scale.

^†^*p* < 0.1, ^*^p < 0.05, ^**^p < 0.01.

### 3.4. Changes in MBG and PCAG between 30 min and 1 week after endoscopy

**Figure 4:**
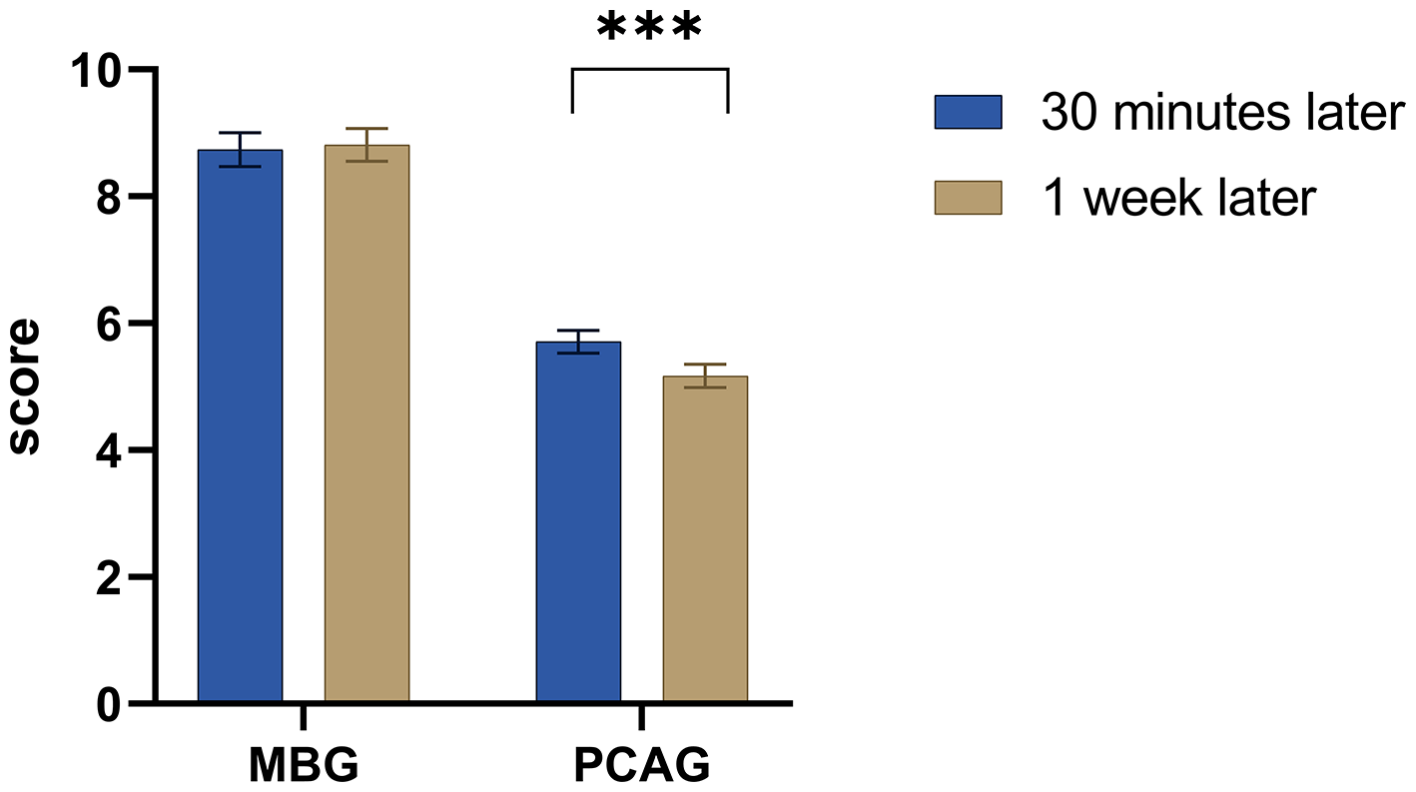
Changes in MBG and PCAG between 30 min and 1 week after endoscopic examination. Paired t-test was adopted. The mean value of PCAG-30 min (5.71) was significantly higher than the mean value of PCAG-1 week (5.17) (*p* < 0.001). However, there was no significant difference between MBG-30 min and MBG-1 week (*t* = −0.464, *p* = 0.643). MBG, Morphine-amphetamine subscale; PCAG, pentobarbital chlorpromazine ethanol subscale. Data were presented as mean ± SEM. ^***^p < 0.001.

In the present study, we found that compared to MBG-30 min, the MBG-1 week did not significantly change. However, the PCAG-1 week was significantly decreased compared with PCAG-30 min (*p* < 0.001) (Figure 4).

### 3.5. Effect of dreaming on euphoria and sedation

**Figure 5:**
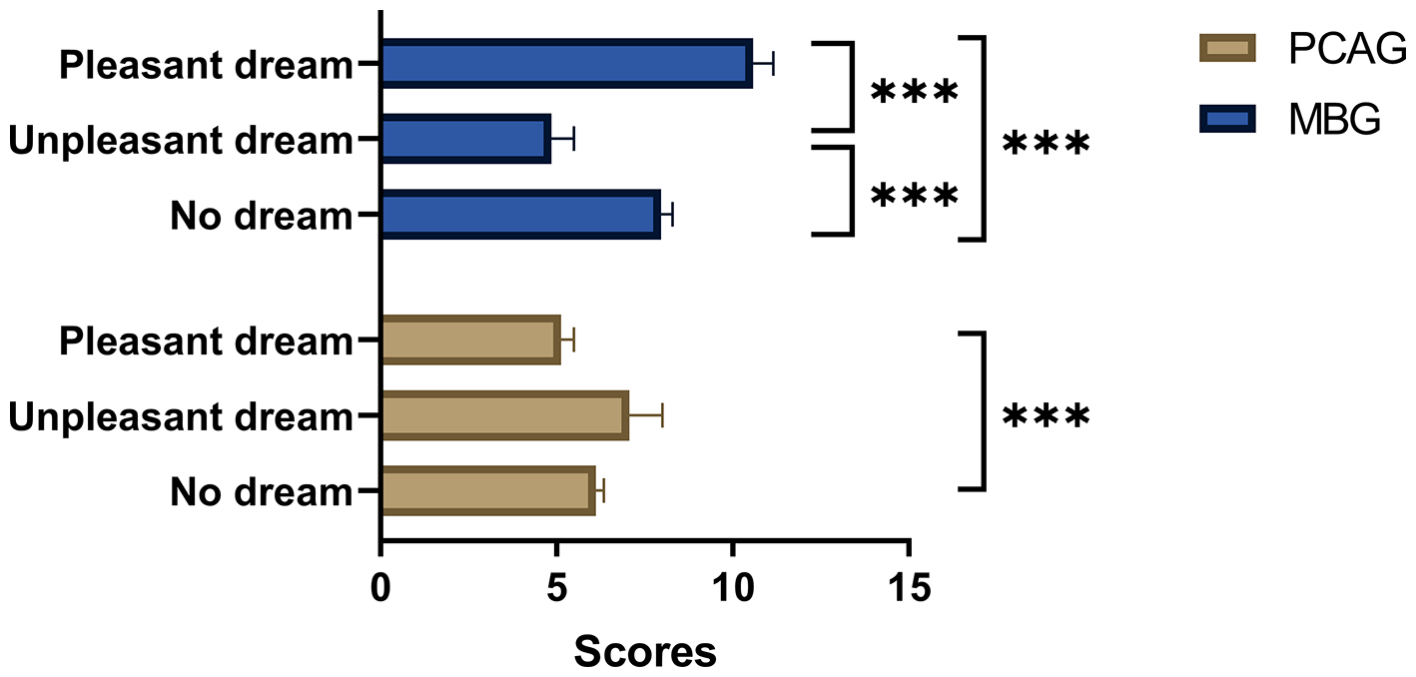
Analysis of MBG/PCAG and dreaming. One way ANOVA was used to study the differences between dream condition for MBG-30 min and PCAG-30 min. There was a significant difference in MBG-30 min between patients who had pleasant dreams, those who had unpleasant dreams, and those who did not dream (*F* = 17.661, *p*<0.001). There was also a significant difference at the 0.05 level in the PCAG-30 min between patients who had pleasant dreams and those who had no dream (*F* = 4.392, *p* = 0.013). MBG: Morphine-amphetamine subscale; PCAG, pentobarbital chlorpromazine ethanol subscale. Data were presented as mean and SEM. ^*^p < 0.05, ^***^p < 0.001.

To determine the subjective and emotional effects of propofol, we recorded whether the subjects dreamed and whether their dreams were pleasant. To further study the effect of dreaming on the euphoria and sedation index, an analysis of dreaming was conducted (Table 5 and Figure 5). Dreams had a significant effect on MBG-30 min (*p* < 0.001) and PCAG-30 min values (*p* = 0.013). We further analyzed the associations between the presence of dreams, the condition of dreams (pleasant or not), and the various possible influencing factors (Supplementary Table S1). Different dream conditions showed significant differences for MBG-30 min (*p* < 0.001) and PCAG-30 min (*p* = 0.003). In addition, there was a statistical difference in MBG-30 min scores (*p* < 0.001), PCAG-30 min scores (*p* = 0.003), propofol dose (*p* = 0.048), and total propofol infusion (*p* = 0.008) between dreamers and non-dreamers (Supplementary Table S1). The MBG-30 min score of dreamers was 9.97, whereas that of non-dreamers was 8.07 (*p* < 0.001). The MBG-30 min score of dreamers with pleasant dreams was 10.76, whereas that of dreamers with unpleasant dreams was 7.89 (*p* < 0.001) (Supplementary Table S1). Moreover, dreams had a significant effect on MBG-1 week (*p* < 0.001) but not on PCAG-1 week (*p* = 0.83) (Supplementary Table S2). This is remarkable, as it showed that dreaming had an effect on euphoria memory but not on sedation.

**Table 5:** Analysis of MBG/PCAG and dreaming.

Items	Categories	*n*	Mean	Std. Deviation	*p*
MBG-30 min	No dream	246	8.07	4.63	0.000***
	Not pleasant dream	16	5.19	2.90	
	Pleasant dream	98	10.76	4.31	
	Total	360	8.67	4.69	
PCAG-30 min	No dream	246	6.06	3.22	0.013*
	Not pleasant dream	16	6.69	3.52	
	Pleasant dream	98	5.03	2.88	
	Total	360	5.81	3.17	

Values are expressed as mean ± standard deviation (SD). The Chi2 square test was performed to compare the difference between groups. ^*^p < 0.05, ***p  < 0.001.

### 3.6. Effect of other drugs used in anesthesia on euphoria or sedation

To further investigate the effect of etomidate on euphoria, we performed a subgroup analysis on MBG and PCAG in patients with or without etomidate. Unexpectedly, the use of etomidate significantly reduced MBG-30 min (*p* = 0.005) and MBG-1 week (*p* < 0.001) as well as significantly increased PCAG-30 min (*p* = 0.001) and PCAG-1 week (*p* = 0.001) (Table 6).

**Table 6:** Differences between whether to inject etomidate.

	Use etomidate or not (Mean ± Std. deviation)		*F*	*p*
	No etomidate (*n* = 181)	Etomidate (*n* = 179)		
MBG-30 min	9.36 ± 4.85	7.97 ± 4.42	8.167	0.005^**^
PCAG-30 min	5.28 ± 3.17	6.34 ± 3.10	10.389	0.001^**^
MBG-1 week	9.96 ± 4.57	7.68 ± 4.33	20.433	0.000^***^
PCAG-1 week	4.58 ± 3.16	5.75 ± 3.24	10.488	0.001^**^

Values are expressed as mean ± standard deviation (SD). The One-way ANOVA test was performed to compare the difference between groups.

^**^p < 0.01, ^***^p < 0.001.

To further analyze the contribution of fentanyl and sufentanil to the postoperative euphoria index or postoperative sedation index, we compared the patients who received fentanyl and those who received sufentanil. A total of 356 of the included participants received either fentanyl or sufentanil infusions, of which 112 received only sufentanil for pain relief, and 244 received only fentanyl. Unexpectedly we did not observe any effect of fentanyl and sufentanil on euphoria (all *p*>0.05) (Supplementary Tables S3, S4).

To determine whether it was the propofol dose and no other drugs that affected the MBG, we divided the patients into two equal-sized groups, HP and LP, according to the propofol dose (mg/kg). The HP group received a higher dose of propofol, whereas the LP group received a lower dose of propofol. Propofol dose groupings were used as study variables. Doses of fentanyl (mcg/kg), sufentanil (mcg/kg), and etomidate (mcg/kg) were featured. Using MBG as the result variable, a 1:1 tendentious score match was performed according to the nearest neighbor method. The standardized deviation of etomidate changed significantly, which means that the matching effect was good. The result are shown in Supplementary Tables S5, S6. As can be seen from the Supplementary Table S6, there was a difference between HP/LP (Higher dose of propofol group/lower dose of propofol group) and MBG before matching (*p* = 0.005) and after matching (*p* = 0.028), which means that the PSM analysis showed a significant difference between HP/LP and MBG.

## 4. Discussion

Propofol is recommended for sedation during gastrointestinal endoscopy, but evidence of its addictive potential has increased (3, 16–18). Additionally, after painful postoperative conditions, patients may have unpleasant emotions that affect their evaluation of their feelings toward propofol (19, 20). Therefore, this study was performed on patients who underwent gastric or gastrointestinal endoscopies, which are associated with minimal pain. In the present research, we included 360 patients undergoing gastric or gastrointestinal endoscopy using propofol as a sedative to assess the euphoric and sedative status at 30 min and 1 week postoperatively and analyze the correlating factors. The results revealed that propofol strongly produced and enhanced patients’ positive moods and increased MBG scores. We found that certain factors such as dreaming, propofol dose, duration of anesthesia, and etomidate dose, may affect this psychiatric effect. However, it is worth noting that some of these risk factors, such as ASA, duration of anesthesia, and fentanyl dose, did not reach conventional levels of statistical significance in our study, although they were close. This may suggest that further research is needed to fully understand the influence of these factors on the development of propofol addiction.

Moreover, we also found that the MBG scale obtained at 1 week of follow-up did not change significantly, but the PCAG was significantly decreased, indicating a longer-lasting memory of euphoria induced by propofol. These findings highlight the potential for propofol to produce long-lasting positive memories, which may contribute to its potential for addiction and abuse. Further research is needed to fully understand the mechanisms behind this phenomenon and to determine the clinical implications of these findings. While the initial euphoria induced by propofol may not persist over time, our findings suggest that the memory of this euphoria may be more prominent. This may be due to the fact that propofol has been shown to affect memory consolidation processes, leading to enhanced memory of events that occurred during propofol administration. It is important to further investigate the mechanisms underlying this phenomenon in order to better understand the potential for propofol addiction and abuse.

MBG is a recognized scale for measuring euphoria, and PCAG is used for sedation (15, 21). In addition, in another study that surveyed 169 patients after gastric endoscopic examination with propofol used as a sedative, the MBG and PCAG scores were 6.3 and 8.1, respectively (16). In contrast, a recent study demonstrated a mean MBG score of only 2.58 in the placebo-controlled group and 3.89 in the cannabis group (22). In previous studies, PCAG was sometimes correlated with MBG depending on the drug description and whether the subjects were the general population or people with a past medical history of drug or alcohol abuse (23). This indicates that the preference for the sedative effect of a drug varies according to individuals (16). Our study measured patients’ feelings toward propofol after a single exposure. Considering that the PCAG in our study was measured around 30 min postoperatively, it could show that the sedative effect of propofol had already disappeared. Therefore, the euphoria caused by propofol was a major factor for drug preference rather than the sedative effect. Given that the sedative effect of propofol, such as sleep induction, disappears quickly and has a decreased residual effect compared with that of other drugs, such as opioids, it had less influence on the mechanism for preference in our study (16).

Our research suggests that dreams may be related to the euphoria induced by propofol. A survey on the use of propofol in healthy people showed that propofol was associated with a significant positive emotional experience (20). Many positive emotional experiences are related to dreams, and the dream content and emotional experience are mostly pleasant. Propofol may also trigger dreams or hallucinations related to sex, which are usually pleasant and vivid (24). The reported cases of dreaming showed more obvious euphoria than those without dreaming, which may be related to the decrease in sexual desire inhibition mediated by propofol (3, 25, 26). Therefore, the addiction to propofol may be related to its hallucinogenic effects. Some patients may wish to experience this pleasant illusion or dream again and seek propofol injection. Moreover, in our study, euphoric subjects had dreams with higher MBG scores and lower PCAG scores. We speculate that residual sedation created difficulties in recalling dreams, resulting in lower MBG scores in patients with higher PCAG.

The opioid analgesics used in the present study, such as low-dose fentanyl or sufentanil, are widely used. However, we did not find that fentanyl or sufentanil affected euphoria or sedation. This may be because the dosage used in endoscopies is not sufficient to cause euphoria. During gastroenteroscopic anesthesia, etomidate is sometimes used for the induction of anesthesia to relieve possible respiratory depression and injection pain. Euphoria has not been reported with etomidate, and we suspect that euphoria may be negatively affected by the lower dose of propofol in combination with etomidate. The results of propensity score matching strongly supported this hypothesis.

Etomidate is a short-acting intravenous anesthetic associated with less respiratory circulatory depression compared with propofol, but its slower rate of metabolism makes it less suitable for maintenance in short operations (27). In gastroenteroscopy, etomidate was used for induction only. At present, no studies have confirmed that etomidate causes euphoria or pleasure, and there are no reports of etomidate addiction cases. Additionally, the slower rate of etomidate metabolism allowed patients to feel more intense residual sedation (27), such as dizziness and weakness, resulting in an elevated PCAG score.

It must be noted that drug euphoria is not addiction. The development of drug addiction requires several steps. Initially, there is a reinforcing effect, such as euphoria, or the reduction of an aversive affective state. In the next step, regular use is established. Eventually, the addictive use of a drug is established, which is characterized by a strong urge even if it causes serious damage to social behavior and health. The risk of transitioning from euphoria to addiction depends on several influencing factors, such as genetic factors and drug availability (28). However, the reminiscence of propofol-induced euphoria is a basic risk factor in the development of drug addiction. This research reminds anesthesiologists and endoscopists to be aware of the psychotropic effects of propofol, especially in patients who repeatedly request gastric or gastrointestinal examinations.

Euphoria is not necessarily negative, and maintaining a certain level of euphoria in clinical practice can improve patient satisfaction with anesthesia and surgery. As a previous study reported, patients who exhibited higher euphoria scores were more likely to report greater satisfaction with their medical experience (17). Their follow-ups were also more cooperative which may be consistent with our follow-up 1 week after the examination.

There are several factors that could potentially influence the outcome of this study. First, patient characteristics such as age, gender, medical history, and mental health status may have impacted the results. To address this, we carefully controlled for these variables in our statistical analyses. Second, the way in which the anesthesia and surgical management was carried out could also have influenced the results. We followed current clinical guidelines and used standardized protocols to ensure consistency in this regard. Third, the research design and statistical analysis methods used in the study could have influenced the results. To mitigate this, we employed propensity score matching (PSM) to control for potential biases and confounders. Finally, external factors such as differences in the healthcare systems or patient populations between centers could have affected the results. This single-center study utilized a research design and statistical analysis that aimed to control for biases and eliminate potential confounders. In order to achieve this, we ensured that our approach to anesthesia management was consistent with clinical guidelines, thus making it more generalizable. Overall, we took a number of steps to minimize the impact of these factors on the study’s results and to ensure the validity and reliability of our findings.

There are also several limitations to the current clinical trial. First, we did not investigate which type of euphoria, such as happiness, feeling high, sedation, dizziness, and light-heartedness (3), occurred for each euphoric individual. It would have been useful to include information about dreams. Although we tried to obtain data regarding whether or not a participant experienced any vivid or unusual dreams while under anesthesia, we were unable to do so due to technical difficulties. Second, we did not investigate the details and types of dreams, so we could not determine what kind of euphoria the dream caused. Third, the bispectral index monitoring was not adopted in our study, making it difficult to study the relationship between depth of anesthesia and euphoric effects. Fourth, the relationship between euphoria and patient satisfaction was not investigated. Last but not least, psychotic effects of propofol were examined using a battery of questioners. Thus, presented results are based on subjective estimations made by investigators. It would not be possible to completely eliminate bias due to subjectivity. Patients were asked briefly whether they had a dream and, if so, whether the dreams were pleasant. However, since the questions were phrased simply as “Did you experience a good dream?” and “Were your dreams pleasurable?,” the answers provided could have easily reflected subjective perceptions of their experiences rather than objective facts.

Our inquiry aimed to uncover the risk factors for propofol addiction amid general anesthesia, such as those in endoscopic procedures where pain is minimal. Our arsenal of measures and procedures encompassed a series of scales, and we enrolled a larger sample size compared to prior studies. With this, we ascertained the euphoric effects of propofol on our subjects and recognized factors correlated with these effects, such as the dose and duration of propofol administered and the presence of additional drugs. Furthermore, our investigation probed how long the euphoria memory persists beyond observation, with our findings highlighting that the euphoria can endure up to a week. Although this does not imply propofol addiction, it warrants further investigations into the matter. In sum, our study provides a significant advancement over earlier research by offering insights into the mechanisms behind propofol’s euphoric effects, potentially informing future research on addiction and abuse of propofol.

In our next study, we plan to utilize more objective measures, such as EEG (electroencephalogram) and EOG (electrooculogram), to evaluate the euphoric state of patients receiving propofol. Additionally, we will investigate other factors that may influence the development of propofol-induced euphoria, such as the type of dreams experienced and blood biomarkers. Additionally, we will conduct our study in multiple centers to enhance the external validity of our findings. By utilizing advanced research designs and techniques, we hope to provide more robust and reliable evidence on the potential for propofol to induce euphoria and addiction.

## 5. Conclusion

Our study suggests that propofol may elicit euphoria, potentially contributing to the development of addiction. Several factors, such as dreaming, propofol dosage, anesthesia duration, and etomidate dosage, were identified as risk factors for propofol addiction. It is imperative to raise awareness of these risks and foster a comprehensive understanding of the association between propofol’s pharmacological properties and addiction development. We urge medical professionals to be vigilant for signs of euphoria during gastric or gastrointestinal endoscopy and to evaluate whether such euphoric experiences may be linked to drug addiction. Our findings underscore the significance of judicious monitoring and cautious consideration of these potential hazards when administering propofol, especially among susceptible individuals. Further investigation is required to fully comprehend propofol addiction potential and devise approaches to counteract this risk. Moreover, physicians ought to be mindful of the possibility that patients may seek repeated gastric or gastrointestinal procedures due to propofol-induced euphoria.

## Data availability statement

The raw data supporting the conclusions of this article will be made available by the authors, without undue reservation.

## Ethics statement

The studies involving human participants were reviewed and approved by The Ethics Committee of Peking University Third Hospital, Peking University Third Hospital. The patients/participants provided their written informed consent to participate in this study.

## Author contributions

XG and MX contributed to the conception of the study. JY instructed psychological evaluation. YH contributed significantly to analysis and manuscript preparation. KZ and NY carried out the observation and wrote the manuscript. XW, NK, and TZ helped perform the analysis with constructive discussions. All authors contributed to the article and approved the submitted version.

## Funding

This work was supported by Bethune Charitable Foundation, Wu Jieping Medical Foundation (320.6750.2020-21-3), the National Natural Science Foundation of China (81901095), and Interdisciplinary Medicine Seed Fund of Peking University (BMU2021MX026).

## Conflict of interest

The authors declare that the research was conducted in the absence of any commercial or financial relationships that could be construed as a potential conflict of interest.

## Publisher’s note

All claims expressed in this article are solely those of the authors and do not necessarily represent those of their affiliated organizations, or those of the publisher, the editors and the reviewers. Any product that may be evaluated in this article, or claim that may be made by its manufacturer, is not guaranteed or endorsed by the publisher.
