# Supplementary material for: Factors associated with euphoria in a large subset of cases using propofol sedation during gastrointestinal endoscopy
Source: Front Psychiatry. 2023 Apr 27;14:1001626. doi: 10.3389/fpsyt.2023.1001626 (PMC10174461; doi:10.3389/fpsyt.2023.1001626)
Supplement: Supplementary file 1 [file Data_Sheet_1.docx]

**informed consent**

(Prospective cohort study of non-narcotic effects of propofol in patients undergoing gastrointestinal endoscopy)

You are invited to take part in this study because you are eligible for study entry. Your research doctor or researcher will fully explain the content of the informed consent form to you. Please read this informed consent form carefully and make a prudent decision about whether to participate in the study. If you are participating in another study, please tell your study doctor or research staff.

The content/nature, risks, and other important information of this study are as follows:

# 1. Why is this study being done?

# Propofol is currently the most widely used ultra-short-acting intravenous anesthetic in clinical practice, and its effects on mood, affective and cognitive functions have attracted much attention in recent years. The results of international and previous studies of our research group all suggest that the administration of propofol can lead to a significant increase in the level of dopamine in the nucleus accumbens, which may lead to changes in mood, emotion, and cognitive function.

# In this study, patients who planned to undergo painless gastrointestinal endoscopy were selected, and the corresponding smoking and drinking conditions, anxiety and depression status, and sleep conditions were scored before the operation, and the anesthesia and operation conditions during the operation were observed, and recorded. You will be asked about any propofol-related specific feelings on Day 1 and Day 30 to assess these effects of propofol.

# research population

This study intends to include 360 subjects.

**Inclusion criteria:** ASA grade 1-2 adult patients undergoing painless gastroscopy in our hospital

**Exclusion criteria: ①** Refusal to sign the informed consent form**; ②** Previous cognitive dysfunction, any history of neuropsychiatric diseases (such as depression), and any history of drug abuse**; ③** Drinking tea, drinking alcohol, and taking stimulants within a week**; ④** Language disorders, mental disorders Blurredness, drowsiness, coma, etc. cannot be evaluated before operation**; ⑤ Allergy** to propofol or adverse reactions such as propofol intolerance in the past

# 3. What is included in this study?

This study is observational.

**Research Process**

If you agree to participate in this study and sign the informed consent form, you will undergo test-related inspections and procedures according to the protocol to confirm whether you are suitable for participating in this study.

Before the operation, the anesthesiologist will give you a score for anxiety, etc. in the ward or the waiting area of the outpatient clinic.

After entering the operating room, non-invasive blood pressure, blood oxygen saturation, and heart rate monitoring were performed according to routine requirements.

Routine anesthesia and surgery are then performed.

Thirty minutes after surgery, on days 7 and 30, a trained physician conducts a detailed survey interview by phone or in-person to measure your emotional response to the anesthetic and changes in cognitive function.

# 4. What are the risks of taking part in this study?

This study is an observational study with interviews as the main method. During the whole process of the study, no intervention will be made in the medical process. Except for collecting and recording routine monitoring data of anesthesia operations, no additional tests or inspections will be carried out. There are no additional risks to you from taking part in this research study. If you have any discomfort during the study, whether it is related to the study or not, please contact your doctor in time. Also, you can stop at any time during the research process. You may opt-out of the study at any time without losing any benefits you would have received.

# 5. What are the benefits of participating in research?

If you agree to take part in this research study, you may not receive the direct medical benefit. But we hope that the information gained from your participation in this research will help the development of medicine in the future and produce obvious social benefits. If you do not take part in this study, you will still receive standardized anesthesia management.

# 6. Will my information be kept confidential?

We will keep your research records confidential as required by law. The relevant laws of our country provide guarantees for the security of privacy, data, and authorized access. Unless required by relevant laws, your name, ID number, address, telephone number, or any information that can directly identify you in the research records will not be disclosed outside Peking University Third Hospital. For those research information about you that is transmitted outside Peking University Third Hospital, we will use a unique number to represent you, and the coded information will be properly stored in Peking University Third Hospital. When publishing research information and data obtained from this study at scientific conferences or scientific journals, your identity will not be disclosed.

# 7. What about research fees?

This study is an observational study with interviews as the main method. During the whole process of the study, no intervention will be made in the medical process. In addition to collecting and recording routine monitoring data of anesthesia operations, no additional tests or inspections will be carried out. Therefore, participating in this study will not increase the subject's additional costs beyond routine treatment/examination/operation, and will not receive direct financial compensation.

# 8. In the event of a study-related injury

This study is an observational study with interviews as the main method. During the whole process of the study, no intervention will be made in the medical process. Except for collecting and recording routine monitoring data of anesthesia operations, no additional tests or inspections will be carried out. Therefore, no research-related injury will occur in this study.

# 9. Refusal to participate in or withdraw from the study

Your participation in the trial is voluntary, and you can refuse to participate or withdraw from the trial in any way at any stage without being discriminated against or retaliated against, and your medical treatment and rights will not be affected.

If you have a serious adverse reaction, or if your study doctor feels that it is not in your best interest to continue participating in the study, he or she will decide to withdraw you from the study. If this happens, we will keep you informed and your study doctor will discuss your other options with you.

# 10. Related inquiries _

If you have any questions related to this study, please contact Dr. Kaixuan Zhao, Tel/ WeChat 15084764467.

If you have any questions related to your rights, or you want to express your dissatisfaction and worries during the process of participating in this research, please contact the Comprehensive Research Ethics Office of Peking University Third Hospital at 010-82265571.

**Inform statement**

"I have informed the subject of the research background, purpose, steps, risks, and benefits of the Prospective Cohort Study on Propofol's Non-Anesthesia Effects in Patients Undergoing Gastroscopy, and given him/her enough time to read the informed consent book, discussed with others, and answered their questions about the research; I have told the subject that he can contact Zhao Kaixuan at any time when he encounters problems related to the research, and he can always contact Zhao Kaixuan when he encounters problems related to his rights/interests. Contact the Comprehensive Research Ethics Office of Peking University Third Hospital and provide accurate contact information; I have informed that the subject can withdraw from this study at any time without any reason*;* I have informed that the subject will receive this A copy of the informed consent form containing my and his/her signatures."

telephone number of the researcher who obtained the informed consent date

# informed consent statement

"I have been informed of the background, purpose, steps, risks, and benefits of the study " Prospective Cohort Study of Propofol's Non-Anesthetic Effects in Patients Undergoing Gastrointestinal Endoscopy". I have had enough time and opportunity to ask questions. I am satisfied with the answer. I have also been informed whom to contact if I have questions, grievances, concerns, or want further information. I have read this informed consent form, agree to participate in this study, and commit to providing the information, laboratory test results, etc. of the participant are valid. I know that I can withdraw from this study at any time without any reason. I was told that I will get a copy of this informed consent form, which contains the signatures of me and the researcher .”

Subject's signature contact number date
