# Supplementary material for: Factors associated with euphoria in a large subset of cases using propofol sedation during gastrointestinal endoscopy
Source: Front Psychiatry. 2023 Apr 27;14:1001626. doi: 10.3389/fpsyt.2023.1001626 (PMC10174461; doi:10.3389/fpsyt.2023.1001626)
Supplement: Supplementary file 2 [file Table_1.docx]

|  | Dream Condition (Mean±Std. Deviation) | | | | | P1 | | Dream Condition (Mean±Std. Deviation) | | |  | *P2* |
| --- | --- | --- | --- | --- | --- | --- | --- | --- | --- | --- | --- | --- |
|  | Non-dreamers(*n*=246) | Dreamers (*n*=114) | | | |  |  | Non-dreamers and Non-pleasure reported dreamers(*n*=262) | | Pleasure reported dreamers (*n*=98) |  |  |
| Age | 43.57±12.31 | 44.83±13.09 | | | | 0.377 | | 43.57±12.25 | | 45.04±13.36 |  | 0.324 |
| BMI | 23.02±3.32 | 22.54±3.00 | | | | 0.197 | | 23.01±3.37 | | 22.49±2.78 |  | 0.173 |
| Total propofol infusion(mg) | 164.71±76.93 | 186.22±114.56 | | | | 0.037* | | 162.78±78.14 | | 194.89±115.85 |  | 0.003** |
| Propofol infusion rate(mg/kg/min) | 0.16±0.08 | 0.17±0.07 | | | | 0.352 | | 0.16±0.08 | | 0.17±0.07 |  | 0.267 |
| Propofol dosage(mg/kg) | 2.58±1.13 | 3.01±1.98 | | | | 0.503 | | 2.56±1.16 | | 3.14±2.02 |  | 0.008** |
| Induction dose (mg) | 59.83±35.00 | 66.30±41.73 | | | | 0.031* | | 59.32±35.41 | | 68.71±41.43 |  | 0.048* |
| Infusion speed （ml/h） | 33.67±10.27 | 33.67±7.28 | | | | 0.152 | | 33.64±10.17 | | 33.73±7.06 |  | 0.933 |
| Sufentanyl dose(μg/kg) | 0.03±0.05 | 0.04±0.05 | | | | 1.000 | | 0.03±0.05 | | 0.04±0.05 |  | 0.571 |
| Fentanyl dose(μg/kg) | 1.09±0.77 | 1.02±0.80 | | | | 0.228 | | 1.07±0.78 | | 1.05±0.79 |  | 0.817 |
| Etomidate dose(mg/kg) | 0.10±0.12 | 0.09±0.10 | | | | 0.396 | | 0.10±0.12 | | 0.08±0.09 |  | 0.242 |
| Smoking | 0.77±3.38 | 1.23±4.68 | | | | 0.558 | | 0.74±3.29 | | 1.38±5.01 |  | 0.248 |
| AUDIT | 1.49±2.74 | 1.25±2.55 | | | | 0.419 | | 1.42±2.68 | | 1.40±2.71 |  | 0.945 |
| BAI | 23.02±3.43 | 23.30±3.53 | | | | 0.480 | | 23.04±3.40 | | 23.30±3.65 |  | 0.530 |
| PSQI | 3.55±3.55 | 3.69±3.99 | | | | 0.739 | | 3.58±3.55 | | 3.62±4.08 |  | 0.932 |
| CESD | 2.32±3.87 | 2.20±3.74 | | | | 0.783 | | 2.33±3.85 | | 2.16±3.78 |  | 0.716 |
| MBG-30min | 8.07±4.63 | 9.97±4.57 | | | | 0.000** | | 7.89±4.59 | | 10.76±4.31 |  | 0.000*** |
| PCAG-30min | 6.06±3.22 | 5.26±3.02 | | | | 0.027* | | 6.10±3.23 | | 5.03±2.88 |  | 0.003** |
| * *p*<0.05 ** *p*<0.01 | | |  |  |  | |  | |  | | | |

Supplementary Table 1. Differences between dreamers and non-dreamers and differences between pleasure dreamers and non-pleasure dreamers. Values are expressed as mean±standard deviation(SD). P1 and P2 are t-test p values of two varieties, respectively, and they were almost identical. * *p*<0.05, ** *p*<0.01, *** *p*<0.001. BMI: Body Mass Index. ASA: American Society of Anesthesiologists Classification (ASA Class). AUDIT: The Alcohol Use Disorders Identification Test. BAI: Beck Anxiety Inventory. PSQI: Pittsburgh Sleep Quality Index. CESD: Center for Epidemiologic Studies Depression Scale.

|  | | | | | |
| --- | --- | --- | --- | --- | --- |
|  | Dream Condition (Mean±Std. Deviation) | | | *F* | *p* |
|  | None dream (*n*=246) | Not Pleasant Dream (*n*=16) | Pleasant Dream(*n*=98) |  |  |
| MBG-1 week | 8.29±4.65 | 5.40±3.22 | 10.64±3.98 | 13.437 | 0.000** |
| PCAG-1week | 5.22±3.33 | 4.73±2.91 | 5.10±3.14 | 0.181 | 0.834 |
| * *p*<0.05 ** *p*<0.01 | | | | | |

Supplementary Table 2. Analysis of MBG/PCAG and dreaming. Data were presented as mean and SEM. * p<0.05, *** p<0.001.

|  | Use Fentanyl or Sufentanil (Mean±Std. Deviation) | | *F* | *p* |
| --- | --- | --- | --- | --- |
|  | Sufentanil ( *n* =112) | Fentanyl ( *n* =244 ) |  |  |
| MBG-30min | 8.51±4.76 | 8.70±4.67 | 0.130 | 0.719 |
| PCAG-30min | 6.18±3.29 | 5.67±3.11 | 1.984 | 0.160 |
|  | | | | |

Supplementary Table 3. Differences between use fentanyl or sufentanil. Values are expressed as mean±standard deviation (SD).

|  | | |  |
| --- | --- | --- | --- |
|  |  | Sufentanyl dose(μg/kg) | Fentanyl dose（μg/kg） |
| MBG-30min | Coefficient | -0.085 | -0.043 |
|  | *p* value | 0.375 | 0.500 |
| PCAG-30min | Coefficient | 0.046 | 0.070 |
|  | *p* value | 0.633 | 0.280 |
|  | | |  |

Supplementary Table 4. Differences due to sufentanil/Fentanyl dose. Values are expressed as mean±standard deviation (SD).

| Item | State | HP (Higher dose of propofol group) | LP(Lower dose of propofol group) | Standardized deviation (%) | Standardized deviation reduction (%) | t value | p-value |
| --- | --- | --- | --- | --- | --- | --- | --- |
| Sufentanil (mcg/kg) | Before match | 0.033 | 0.032 | 1.36% | -201.66% | 0.129 | 0.897 |
|  | After matching | 0.033 | 0.035 | -4.11% |  | -0.391 | 0.696 |
| Fentanyl (mcg/kg) | Before match | 1.068 | 1.065 | 0.45% | -415.01% | 0.043 | 0.966 |
|  | After matching | 1.068 | 1.050 | 2.33% |  | 0.222 | 0.825 |
| Etomidate dose(mg/kg) | Before match | 0.061 | 0.125 | -58.05% | 99.45% | -5.507 | 0.000 |
|  | After matching | 0.061 | 0.062 | -0.32% |  | -0.030 | 0.976 |

Supplementary Table 5. PSM Parallel Hypothesis Testing.

| Item | Treated (experimental group) | Control (control group) | Difference (difference/ATT effect value) | Standard Error Std. Error | t value | p-value |
| --- | --- | --- | --- | --- | --- | --- |
| Unmatched before matching (when all samples) | 9.354 | 7.978 | 1.376 | 0.489 | 2.811 | 0.005 |
| ATT effect | 9.354 | 8.249 | 1.105 | 0.501 | 2.205 | 0.028 |

Supplementary Table 6. ATT effect analysis.
